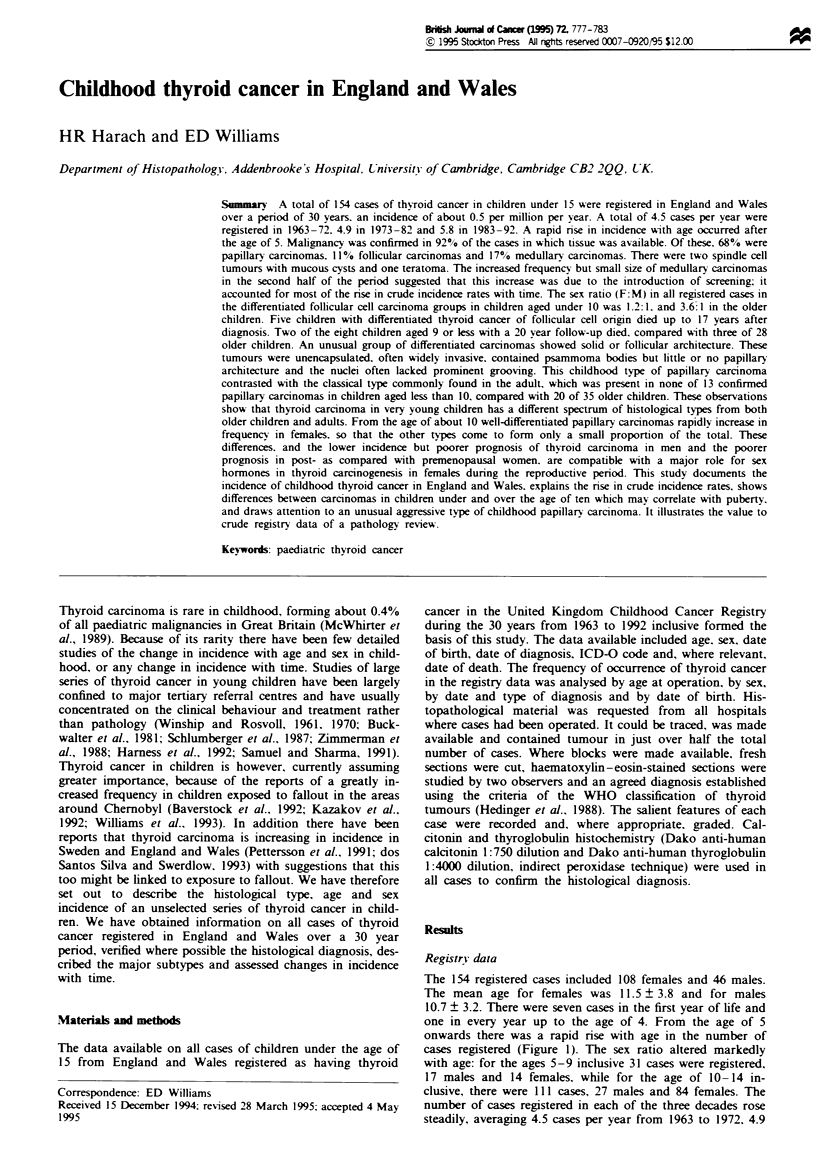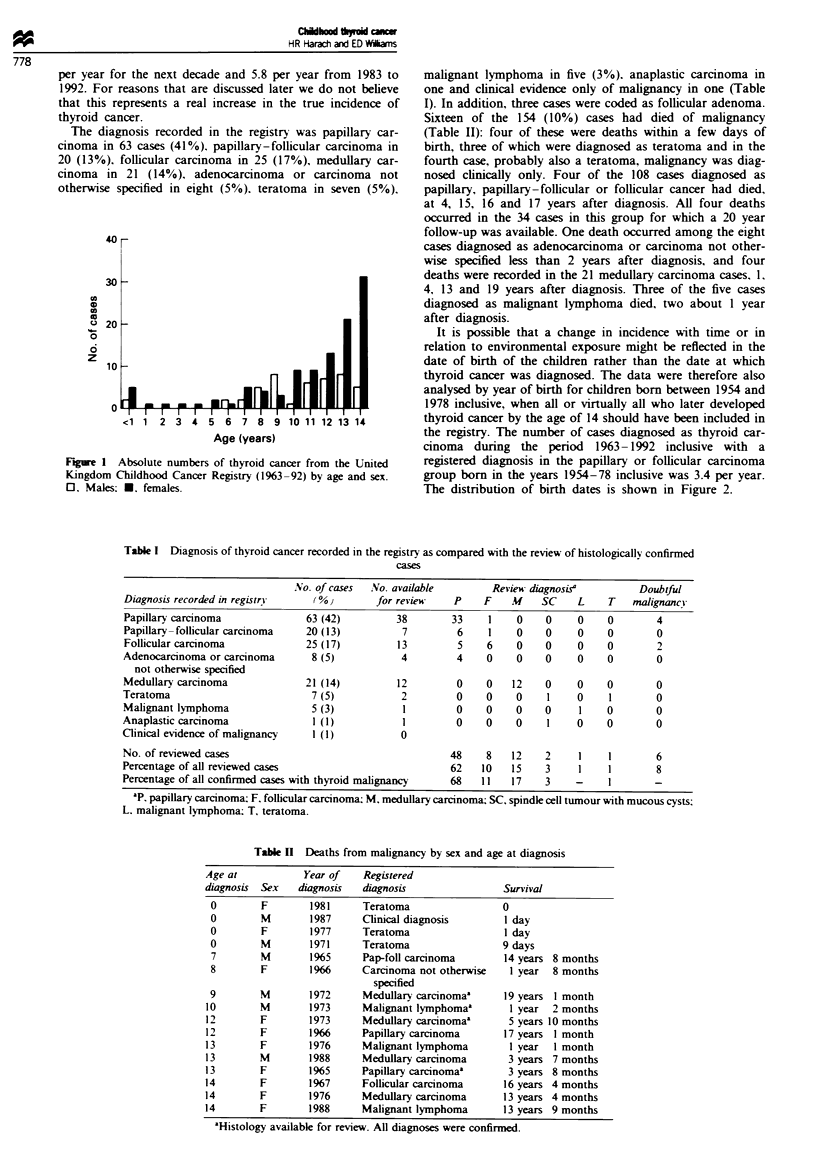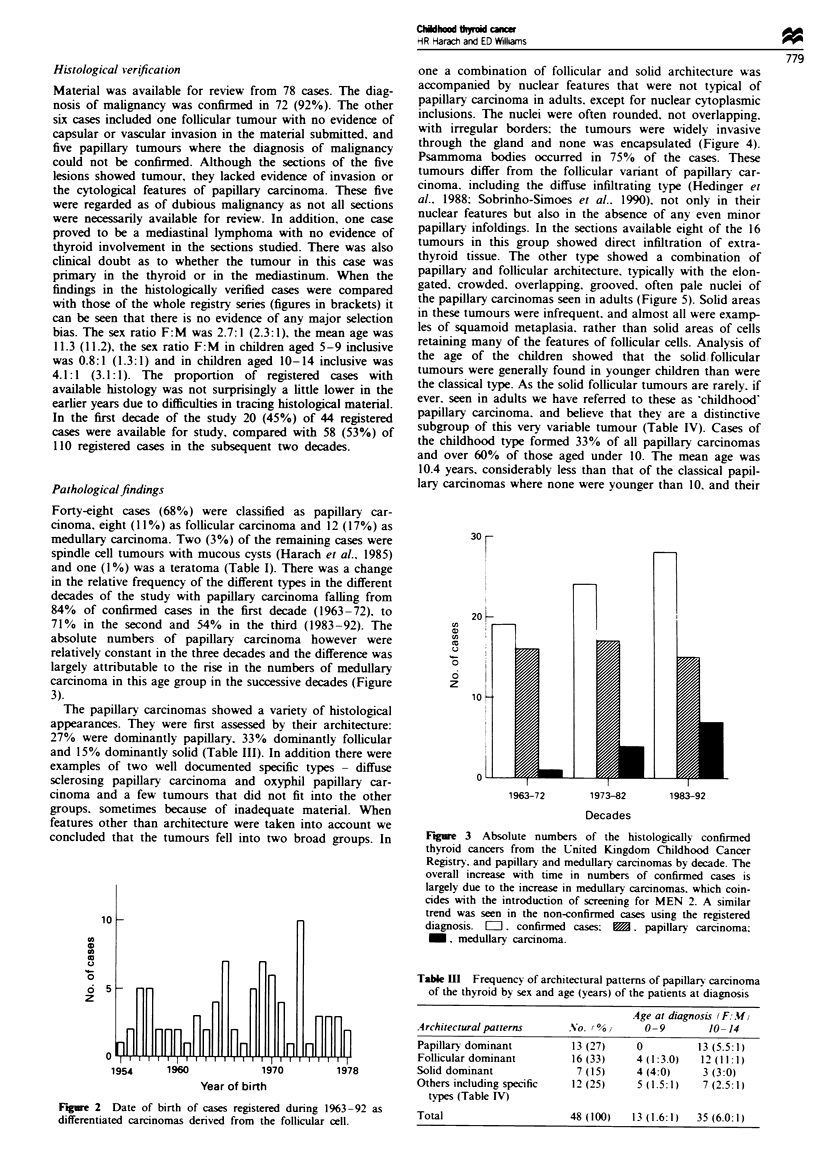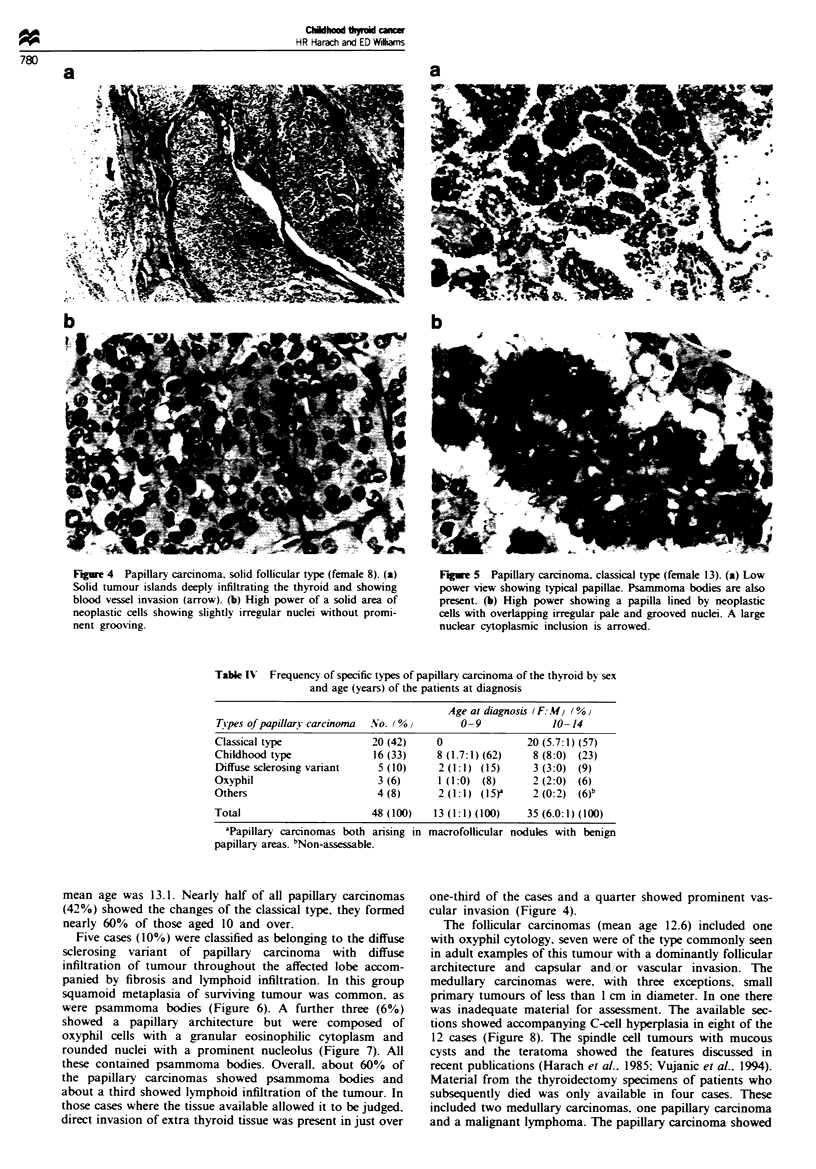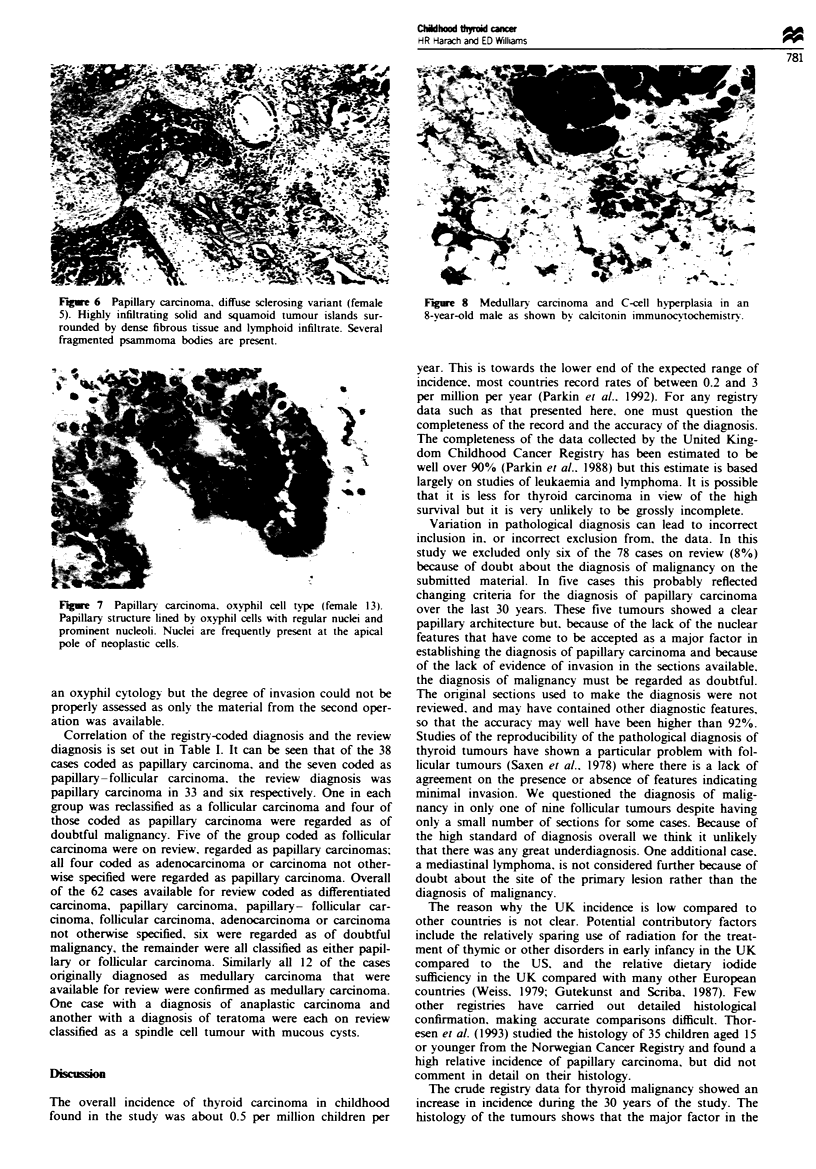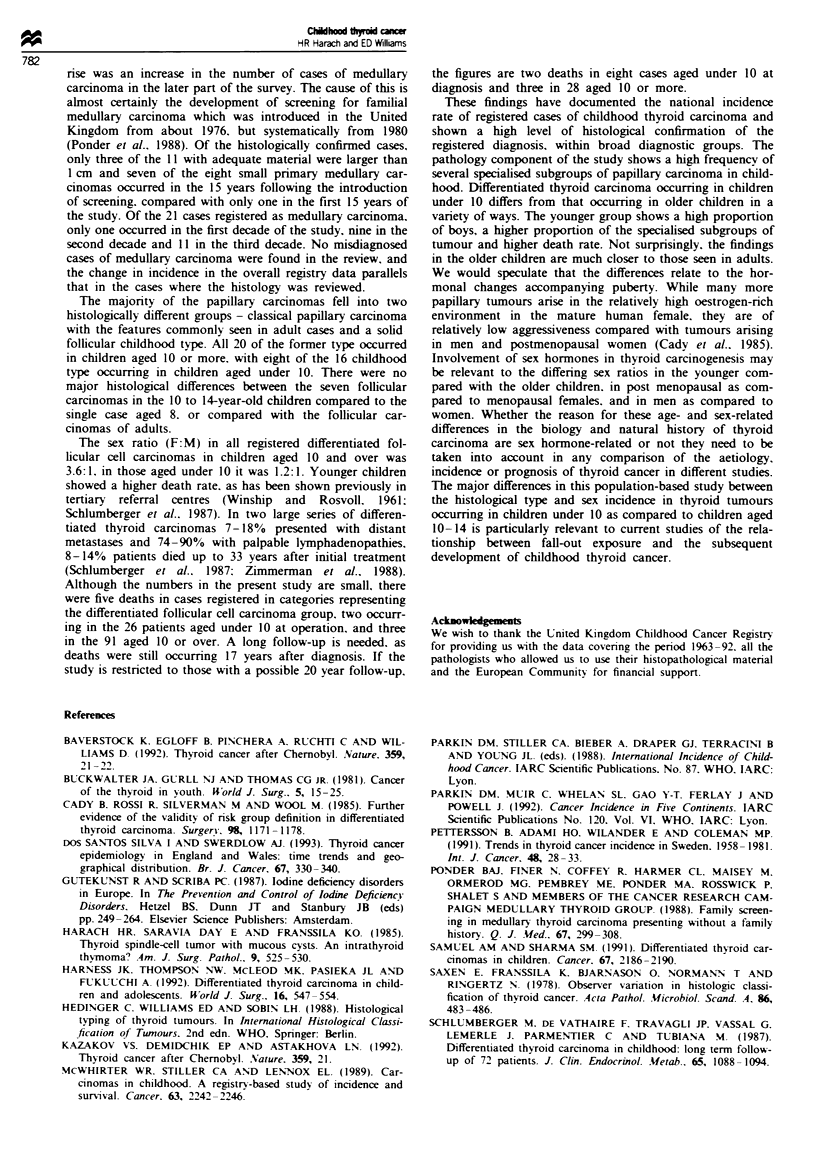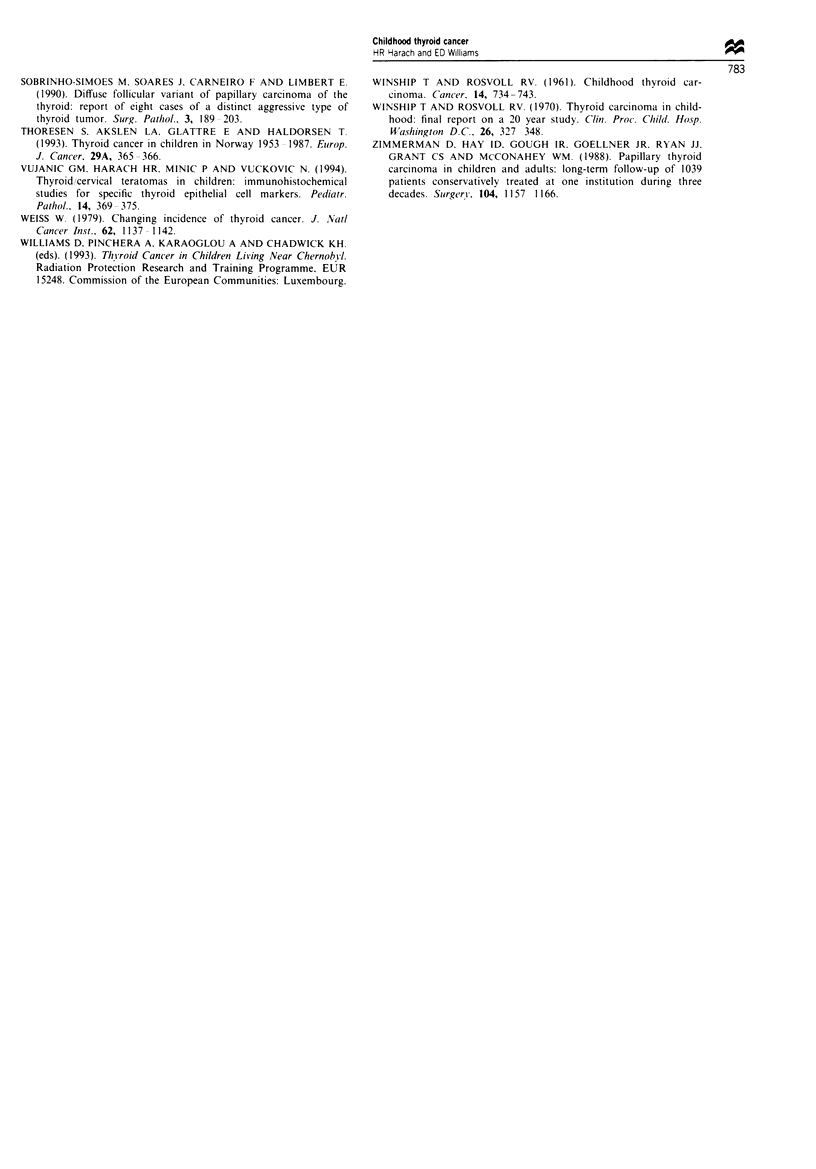# Childhood thyroid cancer in England and Wales.

**DOI:** 10.1038/bjc.1995.410

**Published:** 1995-09

**Authors:** H. R. Harach, E. D. Williams

**Affiliations:** Department of Histopathology, Addenbrooke's Hospital, University of Cambridge, UK.

## Abstract

**Images:**


British Journal of Cancer (1995) 72, 777-783

© 1995 Stockton Press All rights reserved 0007-0920/95 $12.00

### Childhood thyroid cancer in England and Wales

HR Harach and ED Williams

*Department of Histopathology, Addenbrooke's Hospital, University of Cambridge, Cambridge CB2 2QQ, UK.*

**Summary** A total of 154 cases of thyroid cancer in children under 15 were registered in England and Wales over a period of 30 years, an incidence of about 0.5 per million per year. A total of 4.5 cases per year were registered in 1963-72, 4.9 in 1973-82 and 5.8 in 1983-92. A rapid rise in incidence with age occurred after the age of 5. Malignancy was confirmed in 92% of the cases in which tissue was available. Of these, 68% were papillary carcinomas, 11% follicular carcinomas and 17% medullary carcinomas. There were two spindle cell tumours with mucous cysts and one teratoma. The increased frequency but small size of medullary carcinomas in the second half of the period suggested that this increase was due to the introduction of screening; it accounted for most of the rise in crude incidence rates with time. The sex ratio (F:M) in all registered cases in the differentiated follicular cell carcinoma groups in children aged under 10 was 1.2:1, and 3.6:1 in the older children. Five children with differentiated thyroid cancer of follicular cell origin died up to 17 years after diagnosis. Two of the eight children aged 9 or less with a 20 year follow-up died, compared with three of 28 older children. An unusual group of differentiated carcinomas showed solid or follicular architecture. These tumours were unencapsulated, often widely invasive, contained psammoma bodies but little or no papillary architecture and the nuclei often lacked prominent grooving. This childhood type of papillary carcinoma contrasted with the classical type commonly found in the adult, which was present in none of 13 confirmed papillary carcinomas in children aged less than 10, compared with 20 of 35 older children. These observations show that thyroid carcinoma in very young children has a different spectrum of histological types from both older children and adults. From the age of about 10 well-differentiated papillary carcinomas rapidly increase in frequency in females, so that the other types come to form only a small proportion of the total. These differences, and the lower incidence but poorer prognosis of thyroid carcinoma in men and the poorer prognosis in post- as compared with premenopausal women, are compatible with a major role for sex hormones in thyroid carcinogenesis in females during the reproductive period. This study documents the incidence of childhood thyroid cancer in England and Wales, explains the rise in crude incidence rates, shows differences between carcinomas in children under and over the age of ten which may correlate with puberty, and draws attention to an unusual aggressive type of childhood papillary carcinoma. It illustrates the value to crude registry data of a pathology review.

**Keywords**: paediatric thyroid cancer

Thyroid carcinoma is rare in childhood, forming about 0.4% of all paediatric malignancies in Great Britain (McWhirter *et al.*, 1989). Because of its rarity there have been few detailed studies of the change in incidence with age and sex in childhood, or any change in incidence with time. Studies of large series of thyroid cancer in young children have been largely confined to major tertiary referral centres and have usually concentrated on the clinical behaviour and treatment rather than pathology (Winship and Rosvoll, 1961, 1970; Buckwalter *et al.*, 1981; Schlumberger *et al.*, 1987; Zimmerman *et al.*, 1988; Harness *et al.*, 1992; Samuel and Sharma, 1991). Thyroid cancer in children is however, currently assuming greater importance, because of the reports of a greatly increased frequency in children exposed to fallout in the areas around Chernobyl (Baverstock *et al.*, 1992; Kazakov *et al.*, 1992; Williams *et al.*, 1993). In addition there have been reports that thyroid carcinoma is increasing in incidence in Sweden and England and Wales (Pettersson *et al.*, 1991; dos Santos Silva and Swerdlow, 1993) with suggestions that this too might be linked to exposure to fallout. We have therefore set out to describe the histological type, age and sex incidence of an unselected series of thyroid cancer in children. We have obtained information on all cases of thyroid cancer registered in England and Wales over a 30 year period, verified where possible the histological diagnosis, described the major subtypes and assessed changes in incidence with time.

### Materials and methods

The data available on all cases of children under the age of 15 from England and Wales registered as having thyroid cancer in the United Kingdom Childhood Cancer Registry during the 30 years from 1963 to 1992 inclusive formed the basis of this study. The data available included age, sex, date of birth, date of diagnosis, ICD-O code and, where relevant, date of death. The frequency of occurrence of thyroid cancer in the registry data was analysed by age at operation, by sex, by date and type of diagnosis and by date of birth. Histopathological material was requested from all hospitals where cases had been operated. It could be traced, was made available and contained tumour in just over half the total number of cases. Where blocks were made available, fresh sections were cut, haematoxylin-eosin-stained sections were studied by two observers and an agreed diagnosis established using the criteria of the WHO classification of thyroid tumours (Hedinger *et al.*, 1988). The salient features of each case were recorded and, where appropriate, graded. Calcitonin and thyroglobulin histochemistry (Dako anti-human calcitonin 1:750 dilution and Dako anti-human thyroglobulin 1:4000 dilution, indirect peroxidase technique) were used in all cases to confirm the histological diagnosis.

### Results

### *Registry data*

The 154 registered cases included 108 females and 46 males. The mean age for females was 11.5 ± 3.8 and for males 10.7 ± 3.2. There were seven cases in the first year of life and one in every year up to the age of 4. From the age of 5 onwards there was a rapid rise with age in the number of cases registered (Figure 1). The sex ratio altered markedly with age: for the ages 5-9 inclusive 31 cases were registered, 17 males and 14 females, while for the age of 10-14 inclusive, there were 111 cases, 27 males and 84 females. The number of cases registered in each of the three decades rose steadily, averaging 4.5 cases per year from 1963 to 1972, 4.9

Correspondence: ED Williams
Received 15 December 1994; revised 28 March 1995; accepted 4 May 1995

per year for the next decade and 5.8 per year from 1983 to 1992. For reasons that are discussed later we do not believe that this represents a real increase in the true incidence of thyroid cancer.

The diagnosis recorded in the registry was papillary carcinoma in 63 cases (41%), papillary-follicular carcinoma in 20 (13%), follicular carcinoma in 25 (17%), medullary carcinoma in 21 (14%), adenocarcinoma or carcinoma not otherwise specified in eight (5%), teratoma in seven (5%), malignant lymphoma in five (3%), anaplastic carcinoma in one and clinical evidence only of malignancy in one (Table I). In addition, three cases were coded as follicular adenoma. Sixteen of the 154 (10%) cases had died of malignancy (Table II): four of these were deaths within a few days of birth, three of which were diagnosed as teratoma and in the fourth case, probably also a teratoma, malignancy was diagnosed clinically only. Four of the 108 cases diagnosed as papillary, papillary-follicular or follicular cancer had died, at 4, 15, 16 and 17 years after diagnosis. All four deaths occurred in the 34 cases in this group for which a 20 year follow-up was available. One death occurred among the eight cases diagnosed as adenocarcinoma or carcinoma not otherwise specified less than 2 years after diagnosis, and four deaths were recorded in the 21 medullary carcinoma cases, 1, 4, 13 and 19 years after diagnosis. Three of the five cases diagnosed as malignant lymphoma died, two about 1 year after diagnosis.

It is possible that a change in incidence with time or in relation to environmental exposure might be reflected in the date of birth of the children rather than the date at which thyroid cancer was diagnosed. The data were therefore also analysed by year of birth for children born between 1954 and 1978 inclusive, when all or virtually all who later developed thyroid cancer by the age of 14 should have been included in the registry. The number of cases diagnosed as thyroid carcinoma during the period 1963-1992 inclusive with a registered diagnosis in the papillary or follicular carcinoma group born in the years 1954-78 inclusive was 3.4 per year. The distribution of birth dates is shown in Figure 2.

**Figure 1** Absolute numbers of thyroid cancer from the United Kingdom Childhood Cancer Registry (1963-92) by age and sex. □, Males; ■, females.

**Table I** Diagnosis of thyroid cancer recorded in the registry as compared with the review of histologically confirmed cases

| *Diagnosis recorded in registry* | *No. of cases (%)* | *No. available for review* | Review diagnosis[a] *P* | *F* | *M* | *SC* | *L* | *T* | *Doubtful malignancy* |
|---|---|---|---|---|---|---|---|---|---|
| Papillary carcinoma | 63 (42) | 38 | 33 | 1 | 0 | 0 | 0 | 0 | 4 |
| Papillary-follicular carcinoma | 20 (13) | 7 | 6 | 1 | 0 | 0 | 0 | 0 | 0 |
| Follicular carcinoma | 25 (17) | 13 | 5 | 6 | 0 | 0 | 0 | 0 | 2 |
| Adenocarcinoma or carcinoma not otherwise specified | 8 (5) | 4 | 4 | 0 | 0 | 0 | 0 | 0 | 0 |
| Medullary carcinoma | 21 (14) | 12 | 0 | 0 | 12 | 0 | 0 | 0 | 0 |
| Teratoma | 7 (5) | 2 | 0 | 0 | 0 | 1 | 0 | 1 | 0 |
| Malignant lymphoma | 5 (3) | 1 | 0 | 0 | 0 | 0 | 1 | 0 | 0 |
| Anaplastic carcinoma | 1 (1) | 1 | 0 | 0 | 0 | 1 | 0 | 0 | 0 |
| Clinical evidence of malignancy | 1 (1) | 0 | | | | | | | |
| No. of reviewed cases | | | 48 | 8 | 12 | 2 | 1 | 1 | 6 |
| Percentage of all reviewed cases | | | 62 | 10 | 15 | 3 | 1 | 1 | 8 |
| Percentage of all confirmed cases with thyroid malignancy | | | 68 | 11 | 17 | 3 | – | 1 | – |

[a]P, papillary carcinoma; F, follicular carcinoma; M, medullary carcinoma; SC, spindle cell tumour with mucous cysts; L, malignant lymphoma; T, teratoma.

**Table II** Deaths from malignancy by sex and age at diagnosis

| *Age at diagnosis* | *Sex* | *Year of diagnosis* | *Registered diagnosis* | *Survival* |
|---|---|---|---|---|
| 0 | F | 1981 | Teratoma | 0 |
| 0 | M | 1987 | Clinical diagnosis | 1 day |
| 0 | F | 1977 | Teratoma | 1 day |
| 0 | M | 1971 | Teratoma | 9 days |
| 7 | M | 1965 | Pap-foll carcinoma | 14 years 8 months |
| 8 | F | 1966 | Carcinoma not otherwise specified | 1 year 8 months |
| 9 | M | 1972 | Medullary carcinoma[a] | 19 years 1 month |
| 10 | M | 1973 | Malignant lymphoma[a] | 1 year 2 months |
| 12 | F | 1973 | Medullary carcinoma[a] | 5 years 10 months |
| 12 | F | 1966 | Papillary carcinoma | 17 years 1 month |
| 13 | F | 1976 | Malignant lymphoma | 1 year 1 month |
| 13 | M | 1988 | Medullary carcinoma | 3 years 7 months |
| 13 | F | 1965 | Papillary carcinoma[a] | 3 years 8 months |
| 14 | F | 1967 | Follicular carcinoma | 16 years 4 months |
| 14 | F | 1976 | Medullary carcinoma | 13 years 4 months |
| 14 | F | 1988 | Malignant lymphoma | 13 years 9 months |

[a]Histology available for review. All diagnoses were confirmed.

*Histological verification*

Material was available for review from 78 cases. The diagnosis of malignancy was confirmed in 72 (92%). The other six cases included one follicular tumour with no evidence of capsular or vascular invasion in the material submitted, and five papillary tumours where the diagnosis of malignancy could not be confirmed. Although the sections of the five lesions showed tumour, they lacked evidence of invasion or the cytological features of papillary carcinoma. These five were regarded as of dubious malignancy as not all sections were necessarily available for review. In addition, one case proved to be a mediastinal lymphoma with no evidence of thyroid involvement in the sections studied. There was also clinical doubt as to whether the tumour in this case was primary in the thyroid or in the mediastinum. When the findings in the histologically verified cases were compared with those of the whole registry series (figures in brackets) it can be seen that there is no evidence of any major selection bias. The sex ratio F:M was 2.7:1 (2.3:1), the mean age was 11.3 (11.2), the sex ratio F:M in children aged 5–9 inclusive was 0.8:1 (1.3:1) and in children aged 10–14 inclusive was 4.1:1 (3.1:1). The proportion of registered cases with available histology was not surprisingly a little lower in the earlier years due to difficulties in tracing histological material. In the first decade of the study 20 (45%) of 44 registered cases were available for study, compared with 58 (53%) of 110 registered cases in the subsequent two decades.

*Pathological findings*

Forty-eight cases (68%) were classified as papillary carcinoma, eight (11%) as follicular carcinoma and 12 (17%) as medullary carcinoma. Two (3%) of the remaining cases were spindle cell tumours with mucous cysts (Harach *et al.*, 1985) and one (1%) was a teratoma (Table I). There was a change in the relative frequency of the different types in the different decades of the study with papillary carcinoma falling from 84% of confirmed cases in the first decade (1963–72), to 71% in the second and 54% in the third (1983–92). The absolute numbers of papillary carcinoma however were relatively constant in the three decades and the difference was largely attributable to the rise in the numbers of medullary carcinoma in this age group in the successive decades (Figure 3).

The papillary carcinomas showed a variety of histological appearances. They were first assessed by their architecture: 27% were dominantly papillary, 33% dominantly follicular and 15% dominantly solid (Table III). In addition there were examples of two well documented specific types – diffuse sclerosing papillary carcinoma and oxyphil papillary carcinoma and a few tumours that did not fit into the other groups, sometimes because of inadequate material. When features other than architecture were taken into account we concluded that the tumours fell into two broad groups. In one a combination of follicular and solid architecture was accompanied by nuclear features that were not typical of papillary carcinoma in adults, except for nuclear cytoplasmic inclusions. The nuclei were often rounded, not overlapping, with irregular borders; the tumours were widely invasive through the gland and none was encapsulated (Figure 4). Psammoma bodies occurred in 75% of the cases. These tumours differ from the follicular variant of papillary carcinoma, including the diffuse infiltrating type (Hedinger *et al.*, 1988; Sobrinho-Simoes *et al.*, 1990), not only in their nuclear features but also in the absence of any even minor papillary infoldings. In the sections available eight of the 16 tumours in this group showed direct infiltration of extra-thyroid tissue. The other type showed a combination of papillary and follicular architecture, typically with the elongated, crowded, overlapping, grooved, often pale nuclei of the papillary carcinomas seen in adults (Figure 5). Solid areas in these tumours were infrequent, and almost all were examples of squamoid metaplasia, rather than solid areas of cells retaining many of the features of follicular cells. Analysis of the age of the children showed that the solid follicular tumours were generally found in younger children than were the classical type. As the solid follicular tumours are rarely, if ever, seen in adults we have referred to these as 'childhood' papillary carcinoma, and believe that they are a distinctive subgroup of this very variable tumour (Table IV). Cases of the childhood type formed 33% of all papillary carcinomas and over 60% of those aged under 10. The mean age was 10.4 years, considerably less than that of the classical papillary carcinomas where none were younger than 10, and their

**Figure 2** Date of birth of cases registered during 1963–92 as differentiated carcinomas derived from the follicular cell.

**Figure 3** Absolute numbers of the histologically confirmed thyroid cancers from the United Kingdom Childhood Cancer Registry, and papillary and medullary carcinomas by decade. The overall increase with time in numbers of confirmed cases is largely due to the increase in medullary carcinomas, which coincides with the introduction of screening for MEN 2. A similar trend was seen in the non-confirmed cases using the registered diagnosis. □, confirmed cases; ▨, papillary carcinoma; ■, medullary carcinoma.

**Table III** Frequency of architectural patterns of papillary carcinoma of the thyroid by sex and age (years) of the patients at diagnosis

| *Architectural patterns* | *No. (%)* | *Age at diagnosis (F:M)* 0–9 | *Age at diagnosis (F:M)* 10–14 |
|---|---|---|---|
| Papillary dominant | 13 (27) | 0 | 13 (5.5:1) |
| Follicular dominant | 16 (33) | 4 (1:3.0) | 12 (11:1) |
| Solid dominant | 7 (15) | 4 (4:0) | 3 (3:0) |
| Others including specific types (Table IV) | 12 (25) | 5 (1.5:1) | 7 (2.5:1) |
| Total | 48 (100) | 13 (1.6:1) | 35 (6.0:1) |

**Figure 4** Papillary carcinoma, solid/follicular type (female 8). (**a**) Solid tumour islands deeply infiltrating the thyroid and showing blood vessel invasion (arrow). (**b**) High power of a solid area of neoplastic cells showing slightly irregular nuclei without prominent grooving.

**Figure 5** Papillary carcinoma, classical type (female 13). (**a**) Low power view showing typical papillae. Psammoma bodies are also present. (**b**) High power showing a papilla lined by neoplastic cells with overlapping irregular pale and grooved nuclei. A large nuclear cytoplasmic inclusion is arrowed.

**Table IV** Frequency of specific types of papillary carcinoma of the thyroid by sex and age (years) of the patients at diagnosis

| *Types of papillary carcinoma* | *No. (%)* | *Age at diagnosis (F:M) (%)* *0–9* | *10–14* |
|---|---|---|---|
| Classical type | 20 (42) | 0 | 20 (5.7:1) (57) |
| Childhood type | 16 (33) | 8 (1.7:1) (62) | 8 (8:0) (23) |
| Diffuse sclerosing variant | 5 (10) | 2 (1:1) (15) | 3 (3:0) (9) |
| Oxyphil | 3 (6) | 1 (1:0) (8) | 2 (2:0) (6) |
| Others | 4 (8) | 2 (1:1) (15)[a] | 2 (0:2) (6)[b] |
| Total | 48 (100) | 13 (1:1) (100) | 35 (6.0:1) (100) |

[a]Papillary carcinomas both arising in macrofollicular nodules with benign papillary areas. [b]Non-assessable.

mean age was 13.1. Nearly half of all papillary carcinomas (42%) showed the changes of the classical type, they formed nearly 60% of those aged 10 and over.

Five cases (10%) were classified as belonging to the diffuse sclerosing variant of papillary carcinoma with diffuse infiltration of tumour throughout the affected lobe accompanied by fibrosis and lymphoid infiltration. In this group squamoid metaplasia of surviving tumour was common, as were psammoma bodies (Figure 6). A further three (6%) showed a papillary architecture but were composed of oxyphil cells with a granular eosinophilic cytoplasm and rounded nuclei with a prominent nucleolus (Figure 7). All these contained psammoma bodies. Overall, about 60% of the papillary carcinomas showed psammoma bodies and about a third showed lymphoid infiltration of the tumour. In those cases where the tissue available allowed it to be judged, direct invasion of extra thyroid tissue was present in just over one-third of the cases and a quarter showed prominent vascular invasion (Figure 4).

The follicular carcinomas (mean age 12.6) included one with oxyphil cytology, seven were of the type commonly seen in adult examples of this tumour with a dominantly follicular architecture and capsular and/or vascular invasion. The medullary carcinomas were, with three exceptions, small primary tumours of less than 1 cm in diameter. In one there was inadequate material for assessment. The available sections showed accompanying C-cell hyperplasia in eight of the 12 cases (Figure 8). The spindle cell tumours with mucous cysts and the teratoma showed the features discussed in recent publications (Harach *et al.*, 1985; Vujanic *et al.*, 1994). Material from the thyroidectomy specimens of patients who subsequently died was only available in four cases. These included two medullary carcinomas, one papillary carcinoma and a malignant lymphoma. The papillary carcinoma showed

**Figure 6** Papillary carcinoma, diffuse sclerosing variant (female 5). Highly infiltrating solid and squamoid tumour islands surrounded by dense fibrous tissue and lymphoid infiltrate. Several fragmented psammoma bodies are present.

**Figure 7** Papillary carcinoma, oxyphil cell type (female 13). Papillary structure lined by oxyphil cells with regular nuclei and prominent nucleoli. Nuclei are frequently present at the apical pole of neoplastic cells.

**Figure 8** Medullary carcinoma and C-cell hyperplasia in an 8-year-old male as shown by calcitonin immunocytochemistry.

an oxyphil cytology but the degree of invasion could not be properly assessed as only the material from the second operation was available.

Correlation of the registry-coded diagnosis and the review diagnosis is set out in Table I. It can be seen that of the 38 cases coded as papillary carcinoma, and the seven coded as papillary–follicular carcinoma, the review diagnosis was papillary carcinoma in 33 and six respectively. One in each group was reclassified as a follicular carcinoma and four of those coded as papillary carcinoma were regarded as of doubtful malignancy. Five of the group coded as follicular carcinoma were on review, regarded as papillary carcinomas; all four coded as adenocarcinoma or carcinoma not otherwise specified were regarded as papillary carcinoma. Overall of the 62 cases available for review coded as differentiated carcinoma, papillary carcinoma, papillary– follicular carcinoma, follicular carcinoma, adenocarcinoma or carcinoma not otherwise specified, six were regarded as of doubtful malignancy, the remainder were all classified as either papillary or follicular carcinoma. Similarly all 12 of the cases originally diagnosed as medullary carcinoma that were available for review were confirmed as medullary carcinoma. One case with a diagnosis of anaplastic carcinoma and another with a diagnosis of teratoma were each on review classified as a spindle cell tumour with mucous cysts.

### Discussion

The overall incidence of thyroid carcinoma in childhood found in the study was about 0.5 per million children per year. This is towards the lower end of the expected range of incidence, most countries record rates of between 0.2 and 3 per million per year (Parkin *et al.*, 1992). For any registry data such as that presented here, one must question the completeness of the record and the accuracy of the diagnosis. The completeness of the data collected by the United Kingdom Childhood Cancer Registry has been estimated to be well over 90% (Parkin *et al.*, 1988) but this estimate is based largely on studies of leukaemia and lymphoma. It is possible that it is less for thyroid carcinoma in view of the high survival but it is very unlikely to be grossly incomplete.

Variation in pathological diagnosis can lead to incorrect inclusion in, or incorrect exclusion from, the data. In this study we excluded only six of the 78 cases on review (8%) because of doubt about the diagnosis of malignancy on the submitted material. In five cases this probably reflected changing criteria for the diagnosis of papillary carcinoma over the last 30 years. These five tumours showed a clear papillary architecture but, because of the lack of the nuclear features that have come to be accepted as a major factor in establishing the diagnosis of papillary carcinoma and because of the lack of evidence of invasion in the sections available, the diagnosis of malignancy must be regarded as doubtful. The original sections used to make the diagnosis were not reviewed, and may have contained other diagnostic features, so that the accuracy may well have been higher than 92%. Studies of the reproducibility of the pathological diagnosis of thyroid tumours have shown a particular problem with follicular tumours (Saxen *et al.*, 1978) where there is a lack of agreement on the presence or absence of features indicating minimal invasion. We questioned the diagnosis of malignancy in only one of nine follicular tumours despite having only a small number of sections for some cases. Because of the high standard of diagnosis overall we think it unlikely that there was any great underdiagnosis. One additional case, a mediastinal lymphoma, is not considered further because of doubt about the site of the primary lesion rather than the diagnosis of malignancy.

The reason why the UK incidence is low compared to other countries is not clear. Potential contributory factors include the relatively sparing use of radiation for the treatment of thymic or other disorders in early infancy in the UK compared to the US, and the relative dietary iodide sufficiency in the UK compared with many other European countries (Weiss, 1979; Gutekunst and Scriba, 1987). Few other registries have carried out detailed histological confirmation, making accurate comparisons difficult. Thoresen *et al.* (1993) studied the histology of 35 children aged 15 or younger from the Norwegian Cancer Registry and found a high relative incidence of papillary carcinoma, but did not comment in detail on their histology.

The crude registry data for thyroid malignancy showed an increase in incidence during the 30 years of the study. The histology of the tumours shows that the major factor in the rise was an increase in the number of cases of medullary carcinoma in the later part of the survey. The cause of this is almost certainly the development of screening for familial medullary carcinoma which was introduced in the United Kingdom from about 1976, but systematically from 1980 (Ponder *et al.*, 1988). Of the histologically confirmed cases, only three of the 11 with adequate material were larger than 1 cm and seven of the eight small primary medullary carcinomas occurred in the 15 years following the introduction of screening, compared with only one in the first 15 years of the study. Of the 21 cases registered as medullary carcinoma, only one occurred in the first decade of the study, nine in the second decade and 11 in the third decade. No misdiagnosed cases of medullary carcinoma were found in the review, and the change in incidence in the overall registry data parallels that in the cases where the histology was reviewed.

The majority of the papillary carcinomas fell into two histologically different groups – classical papillary carcinoma with the features commonly seen in adult cases and a solid follicular childhood type. All 20 of the former type occurred in children aged 10 or more, with eight of the 16 childhood type occurring in children aged under 10. There were no major histological differences between the seven follicular carcinomas in the 10 to 14-year-old children compared to the single case aged 8, or compared with the follicular carcinomas of adults.

The sex ratio (F:M) in all registered differentiated follicular cell carcinomas in children aged 10 and over was 3.6:1, in those aged under 10 it was 1.2:1. Younger children showed a higher death rate, as has been shown previously in tertiary referral centres (Winship and Rosvoll, 1961; Schlumberger *et al.*, 1987). In two large series of differentiated thyroid carcinomas 7–18% presented with distant metastases and 74–90% with palpable lymphadenopathies, 8–14% patients died up to 33 years after initial treatment (Schlumberger *et al.*, 1987; Zimmerman *et al.*, 1988). Although the numbers in the present study are small, there were five deaths in cases registered in categories representing the differentiated follicular cell carcinoma group, two occurring in the 26 patients aged under 10 at operation, and three in the 91 aged 10 or over. A long follow-up is needed, as deaths were still occurring 17 years after diagnosis. If the study is restricted to those with a possible 20 year follow-up, the figures are two deaths in eight cases aged under 10 at diagnosis and three in 28 aged 10 or more.

These findings have documented the national incidence rate of registered cases of childhood thyroid carcinoma and shown a high level of histological confirmation of the registered diagnosis, within broad diagnostic groups. The pathology component of the study shows a high frequency of several specialised subgroups of papillary carcinoma in childhood. Differentiated thyroid carcinoma occurring in children under 10 differs from that occurring in older children in a variety of ways. The younger group shows a high proportion of boys, a higher proportion of the specialised subgroups of tumour and higher death rate. Not surprisingly, the findings in the older children are much closer to those seen in adults. We would speculate that the differences relate to the hormonal changes accompanying puberty. While many more papillary tumours arise in the relatively high oestrogen-rich environment in the mature human female, they are of relatively low aggressiveness compared with tumours arising in men and postmenopausal women (Cady *et al.*, 1985). Involvement of sex hormones in thyroid carcinogenesis may be relevant to the differing sex ratios in the younger compared with the older children, in post menopausal as compared to menopausal females, and in men as compared to women. Whether the reason for these age- and sex-related differences in the biology and natural history of thyroid carcinoma are sex hormone-related or not they need to be taken into account in any comparison of the aetiology, incidence or prognosis of thyroid cancer in different studies. The major differences in this population-based study between the histological type and sex incidence in thyroid tumours occurring in children under 10 as compared to children aged 10–14 is particularly relevant to current studies of the relationship between fall-out exposure and the subsequent development of childhood thyroid cancer.

**Acknowledgements**
We wish to thank the United Kingdom Childhood Cancer Registry for providing us with the data covering the period 1963–92, all the pathologists who allowed us to use their histopathological material and the European Community for financial support.

**References**

BAVERSTOCK K, EGLOFF B, PINCHERA A, RUCHTI C AND WILLIAMS D. (1992). Thyroid cancer after Chernobyl. *Nature*, **359**, 21–22.

BUCKWALTER JA, GURLL NJ AND THOMAS CG JR. (1981). Cancer of the thyroid in youth. *World J. Surg.*, **5**, 15–25.

CADY B, ROSSI R, SILVERMAN M AND WOOL M. (1985). Further evidence of the validity of risk group definition in differentiated thyroid carcinoma. *Surgery*, **98**, 1171–1178.

DOS SANTOS SILVA I AND SWERDLOW AJ. (1993). Thyroid cancer epidemiology in England and Wales: time trends and geographical distribution. *Br. J. Cancer*, **67**, 330–340.

GUTEKUNST R AND SCRIBA PC. (1987). Iodine deficiency disorders in Europe. In *The Prevention and Control of Iodine Deficiency Disorders*. Hetzel BS, Dunn JT and Stanbury JB (eds) pp. 249–264. Elsevier Science Publishers: Amsterdam.

HARACH HR, SARAVIA DAY E AND FRANSSILA KO. (1985). Thyroid spindle-cell tumor with mucous cysts. An intrathyroid thymoma? *Am. J. Surg. Pathol.*, **9**, 525–530.

HARNESS JK, THOMPSON NW, MCLEOD MK, PASIEKA JL AND FUKUUCHI A. (1992). Differentiated thyroid carcinoma in children and adolescents. *World J. Surg.*, **16**, 547–554.

HEDINGER C, WILLIAMS ED AND SOBIN LH. (1988). Histological typing of thyroid tumours. In *International Histological Classification of Tumours*, 2nd edn. WHO, Springer: Berlin.

KAZAKOV VS, DEMIDCHIK EP AND ASTAKHOVA LN. (1992). Thyroid cancer after Chernobyl. *Nature*, **359**, 21.

MCWHIRTER WR, STILLER CA AND LENNOX EL. (1989). Carcinomas in childhood. A registry-based study of incidence and survival. *Cancer*, **63**, 2242–2246.

PARKIN DM, STILLER CA, BIEBER A, DRAPER GJ, TERRACINI B AND YOUNG JL. (eds). (1988). *International Incidence of Childhood Cancer*. IARC Scientific Publications, No. 87, WHO, IARC: Lyon.

PARKIN DM, MUIR C, WHELAN SL, GAO Y-T, FERLAY J AND POWELL J. (1992). *Cancer Incidence in Five Continents*. IARC Scientific Publications No. 120, Vol. VI, WHO, IARC: Lyon.

PETTERSSON B, ADAMI HO, WILANDER E AND COLEMAN MP. (1991). Trends in thyroid cancer incidence in Sweden, 1958–1981. *Int. J. Cancer*, **48**, 28–33.

PONDER BAJ, FINER N, COFFEY R, HARMER CL, MAISEY M, ORMEROD MG, PEMBREY ME, PONDER MA, ROSSWICK P, SHALET S AND MEMBERS OF THE CANCER RESEARCH CAMPAIGN MEDULLARY THYROID GROUP. (1988). Family screening in medullary thyroid carcinoma presenting without a family history. *Q. J. Med.*, **67**, 299–308.

SAMUEL AM AND SHARMA SM. (1991). Differentiated thyroid carcinomas in children. *Cancer*, **67**, 2186–2190.

SAXEN E, FRANSSILA K, BJARNASON O, NORMANN T AND RINGERTZ N. (1978). Observer variation in histologic classification of thyroid cancer. *Acta Pathol. Microbiol. Scand. A*, **86**, 483–486.

SCHLUMBERGER M, DE VATHAIRE F, TRAVAGLI JP, VASSAL G, LEMERLE J, PARMENTIER C AND TUBIANA M. (1987). Differentiated thyroid carcinoma in childhood: long term follow-up of 72 patients. *J. Clin. Endocrinol. Metab.*, **65**, 1088–1094.

SOBRINHO-SIMOES M, SOARES J, CARNEIRO F AND LIMBERT E. (1990). Diffuse follicular variant of papillary carcinoma of the thyroid: report of eight cases of a distinct aggressive type of thyroid tumor. *Surg. Pathol.*, **3**, 189–203.

THORESEN S, AKSLEN LA, GLATTRE E AND HALDORSEN T. (1993). Thyroid cancer in children in Norway 1953–1987. *Europ. J. Cancer*, **29A**, 365–366.

VUJANIC GM, HARACH HR, MINIC P AND VUCKOVIC N. (1994). Thyroid/cervical teratomas in children: immunohistochemical studies for specific thyroid epithelial cell markers. *Pediatr. Pathol.*, **14**, 369–375.

WEISS W. (1979). Changing incidence of thyroid cancer. *J. Natl Cancer Inst.*, **62**, 1137–1142.

WILLIAMS D, PINCHERA A, KARAOGLOU A AND CHADWICK KH. (eds). (1993). *Thyroid Cancer in Children Living Near Chernobyl*. Radiation Protection Research and Training Programme, EUR 15248. Commission of the European Communities: Luxembourg.

WINSHIP T AND ROSVOLL RV. (1961). Childhood thyroid carcinoma. *Cancer*, **14**, 734–743.

WINSHIP T AND ROSVOLL RV. (1970). Thyroid carcinoma in childhood: final report on a 20 year study. *Clin. Proc. Child. Hosp. Washington D.C.*, **26**, 327–348.

ZIMMERMAN D, HAY ID, GOUGH IR, GOELLNER JR, RYAN JJ, GRANT CS AND McCONAHEY WM. (1988). Papillary thyroid carcinoma in children and adults: long-term follow-up of 1039 patients conservatively treated at one institution during three decades. *Surgery*, **104**, 1157–1166.